# Comparative Transcriptome Analysis Highlights the Role of *NlABCG14* in the Honeydew Production of Virulent Brown Planthoppers (*Nilaparvata lugens* Stål) to Resistant Rice Variety

**DOI:** 10.3390/insects15120992

**Published:** 2024-12-15

**Authors:** Shengli Jing, Mengjia Geng, Bojie Lu, Bing Wu, Yuhan Shao, Chenxi Li, Qingqing Yu, Jingang Xu, Wei Hu, Qingsong Liu, Bin Yu

**Affiliations:** 1 College of Life Sciences, Xinyang Normal University, Xinyang 464000, China; shljing@xynu.edu.cn (S.J.); gmj15303837331@163.com (M.G.); wubing113647@163.com (B.W.); syh15083117015@163.com (Y.S.); lcx15237848534@163.com (C.L.); m18211788993_1@163.com (Q.Y.); xjg157164@163.com (J.X.); 2 Hubei Provincial Key Laboratory for Protection and Application of Special Plant Germplasm in Wuling Area of China, College of Life Sciences, South-Central Minzu University, Wuhan 430074, China; lbj202408@163.com; 3 Guangdong Provincial Key Laboratory of New Technology in Rice Breeding, Rice Research Institute, Guangdong Academy of Agricultural Sciences, Guangzhou 510640, China; 2000whu@163.com; 4 State Key Laboratory of Cotton Bio-Breeding and Integrated Utilization, State Key Laboratory of Crop Stress Adaptation and Improvement, Key Laboratory of Plant Stress Biology, School of Life Sciences, Henan University, Kaifeng 475004, China

**Keywords:** brown planthopper, resistant rice, response mechanism, RNA sequencing, ABC transporters

## Abstract

Brown planthoppers (BPHs, *Nilaparvata lugens* Stål) are a major threat to rice cultivation in Asia, necessitating the development of pest-resistant varieties for effective management. However, the adaptability of BPHs has resulted in the development of virulent populations, such as biotype Y BPHs, which exhibit significant virulence against the rice variety YHY15 that harbors the resistance gene *Bph15*. The various response mechanisms of BPH populations to resistant rice varieties are critical yet underexplored. Via RNA sequencing, the present study identified distinct transcriptional profiles in avirulent (biotype 1) and virulent (biotype Y) BPH nymphs both before and after feeding on YHY15 rice. Our findings revealed differential expression patterns of gene clusters involved in protein synthesis, hydrolysis, fatty acid biosynthesis, metabolism, cuticle composition, and translocation. Further analysis elucidated changes in the expression of genes associated with longevity and structural components of cuticles, highlighting specific disruptions in both biotype 1 and biotype Y BPHs. Moreover, the two biotypes showed differences in the expression level of genes involved in ATP-binding cassette (ABC) transporters. A functional assessment of ABC transporter genes revealed a role of *NlABCG14* in the honeydew production of biotype Y BPHs to YHY15 rice, without impacting their survival and developmental dynamics. These insights deepen our understanding of the mechanisms of virulent BPHs response to resistant rice varieties and highlight potential targets for improving pest management strategies.

## Simple Summary

The brown planthopper (BPH) is a major threat to rice crops in Asia, particularly due to its ability to overcome resistant rice varieties designed to control it. This study aimed to understand the various responses of different virulent BPH populations feeding on resistant rice, focusing on biotype Y, known for its strong virulence against YHY15 rice. By analyzing gene activity in BPH nymphs before and after feeding on YHY15 rice, distinct changes were found in genes related to protein production, metabolism, cuticle structure, and detoxification processes. Key findings highlighted specific genetic pathways involved in regulating longevity, fatty acid biosynthesis/metabolism, cuticle composition, and ABC (ATP-binding cassette) transporters, crucial for BPHs survival and development. Particularly, the role of ABC transporters, such as *NlABCG14*, was identified as essential in biotype Y BPHs’ feeding response on YHY15 rice. These insights provide valuable knowledge for developing improved strategies to manage BPH infestations, ensuring more effective protection of rice crops against this damaging pest.

## 1. Introduction

The brown planthopper (BPH), *Nilaparvata lugens* (Stål) (Hemiptera: Delphacidae), is a major pest that has been causing substantial losses in rice production across Asia for decades [1]. BPHs feed on rice phloem sap by using their piercing–sucking mouthparts. Rice plants infested with a BPH population show various symptoms, such as dwarfing, wilting, browning, and drying. In addition, BPHs act as a vector for viral diseases that can lead to the death of rice plants [2,3].

Cultivating resistant rice varieties is among the most environmentally friendly and effective strategies for managing BPH populations. Throughout the evolutionary competition, rice plants have evolved intricate defense mechanisms against BPHs [4], encompassing both basal defenses and those mediated by resistance (R) genes. To date, approximately 40 major BPH resistance genes have been identified across both cultivated and wild rice species [5]. For instance, the rice variety YHY15 exhibits resistance to BPHs owing to the presence of *Bph15* located on the short arm of chromosome 4. Within the *Bph15* region, four genes coding for jacalin-related lectin proteins and one coding for an LRR protein are identified as candidate genes contributing to this resistance [6,7].

A metabolomics analysis of rice leaf sheaths revealed that BPH infestation boosts fatty acid oxidation, the glyoxylate cycle, gluconeogenesis, and the GABA (gamma-aminobutyric acid) shunt in the susceptible rice variety Taichung Native 1 (TN1) plants. In contrast, in the resistant rice variety YHY15, BPH infestation activates glycolysis and the shikimate metabolic pathway [8]. The shikimate metabolic pathway is vital for the production of secondary metabolites that aid in defense against insect herbivores [9,10,11]. These findings suggest that *Bph15* mediates a resistance response by enhancing the synthesis of secondary metabolites through the shikimate metabolic pathway [8]. Furthermore, comprehensive miRNA and mRNA sequencing studies have demonstrated that miRNA regulation, phytohormone signaling, and defense-related metabolic pathways are key components of the response of YHY15 plants to BPH feeding [12]. In particular, defense-related metabolic pathways promote callose deposition, which hinders continuous phloem sap ingestion by BPHs. This process leads to the accumulation of defensive secondary metabolites, including flavonoids, lignin, lignans, and cinnamic acid amides. These secondary metabolites, as downstream products of the shikimate metabolic pathway [12,13,14], disrupt the normal physiological functions of BPHs and strengthen the plant’s defense mechanisms.

Plants have developed complex defense mechanisms against herbivorous insects, and in response, insects have evolved corresponding bypass strategies. Specific biotypes of BPHs exhibit high virulence, thus overcoming the resistance mediated by major resistance genes in plants. For example, biotype 1 of BPH, which typically shows no virulence against any resistant rice varieties, is widespread across Southeast Asia and primarily damages susceptible rice varieties such as TN1 [15]. However, when reared over an extended period, prolonged rearing on the resistant YHY15 rice variety, which contains the *Bph15* gene, a new biotype, designated as biotype Y BPH, emerged. This biotype demonstrated high virulence and an ability to overcome *Bph15*-mediated resistance [16]. Similarly, after force-feeding 40 generations of local BPH on resistant IR56 rice, which carries the *Bph3* gene, the resulting IR56-BPH population acquired the capability to overcome *Bph3*-mediated resistance [17]. Moreover, the Mudgo BPH population causes significant damage to Mudgo rice plants, which possess the *Bph1* gene [18,19]. Thus, targeted strategies should be developed to curb the survival and reproduction of BPH populations. The dynamic interactions of avirulent and virulent BPH strains with resistant rice varieties provide insightful models for exploring the mechanisms underlying resistance adaptation.

Previous studies have demonstrated that avirulent (biotype 1) and virulent (biotype Y) BPHs exhibit markedly different responses when feeding on YHY15 rice [12,20]. Comprehensive small RNA transcriptome analyses identified 26 miRNAs that vary between these two BPH populations, suggesting a potential role of these miRNAs in regulating adaptability to resistant rice varieties [21]. In addition, bulk segregant RNA sequencing has demonstrated that alterations in lipid metabolism and digestive system pathways are pivotal for the capacity of virulent BPHs to thrive on resistant YHY15 rice. In contrast, genes related to carbohydrate, amino acid, and nucleotide metabolism, as well as those involved in the endocrine system and signal transduction, were found to be upregulated in avirulent BPHs feeding on plants [20]. Furthermore, a metabolomics analysis of honeydew revealed significant decreases in the concentrations of most amino acids in honeydew and an increase in the concentration of urea in BPH populations when BPHs were fed on resistant YHY15 plants compared with when BPHs were fed on TN1 plants [8]. This finding suggests that BPHs feeding on resistant YHY15 plants enhances amino acid absorption [8]. However, the precise mechanisms underlying BPHs feeding response on resistant rice varieties remain poorly understood.

In this study, we employed high-throughput sequencing to analyze the mRNA expression profiles of biotype 1 BPH, representing the avirulent population, and biotype Y BPH, representing the virulent population, when feeding on YHY15 rice seedlings. By combining sequence analysis with functional gene assays, we elucidated the response mechanisms employed by BPHs with varying levels of virulence against resistant rice varieties. The findings of the present study can provide a crucial basis for future genome-wide research into BPH-responsive genes and investigations into the adaptability of BPHs to *Bph15*-mediated resistance. Moreover, these findings can enhance our understanding of the complex interactions between rice and BPH, offering valuable insights that could inform the development of effective strategies for managing BPH populations.

## 2. Materials and Methods

### 2.1. Plant and Insect Materials

In this study, we utilized two rice cultivars: Taichung Native 1 (TN1), a susceptible variety, and YHY15, a resistant variety carrying the *Bph15* resistance gene [6]. These were used to rear two populations of BPH, designated as biotype 1 and biotype Y. Biotype 1, an avirulent population, was maintained on TN1 rice. In contrast, a virulent biotype Y population was developed through the adaptation of biotype 1 on YHY15 plants starting in January 2007, over the course of more than 160 generations (samples were collected in July 2019) [16]. All rice plants were cultivated from seeds sown in sponges with dimensions of 6 cm in diameter and 2 cm in height, with six plants per cup. These plants were grown in a controlled-environment incubator maintained at 30 ± 2 °C during the day (16 h, 06:00–22:00) and 28 ± 2 °C at night (8 h, 22:00–06:00). Depending on the experimental requirements, the rice plants were grown for approximately 2 to 5 weeks post-sowing. The BPH populations were maintained at Xinyang Normal University, China, under conditions of 26 ± 1 °C with a 16-h light/8-h dark cycle. Third-instar BPH nymphs were utilized for both the feeding and micro-injection experiments.

### 2.2. BPH Feeding and Sample Collection

Prior to the feeding treatment, the third-instar nymphs underwent a starvation period of 1 h. Post-starved, third-instar nymphs of both biotype 1 and biotype Y were introduced onto three-week-old YHY15 rice plants for feeding. On average, each rice seedling hosted approximately 15 insect individuals. For RNA-seq analysis, nymphs were collected at 0 h as non-feeding controls, representing post-starved insects, and during early (6 h) and late (48 h) feeding periods. Samples collected from non-feeding biotype 1 BPH were designated as ‘T0’, while those feeding on YHY15 rice for 6 or 48 h were labeled as ‘T6’ and ‘T48’, respectively. Similarly, samples from non-feeding biotype Y BPH were designated as ‘Y0’, and those feeding for 6 or 48 h were labeled as ‘Y6’ and ‘Y48’, respectively. All groups of nymphs were snap-frozen in liquid nitrogen and stored at −80 °C for future analysis. Each treatment was conducted with three biological replicates.

### 2.3. RNA Transcriptome Sequencing and Analysis

#### 2.3.1. RNA Extraction, Quantification, and Qualification

Following the manufacturer’s instructions, RNA Trizol reagent was used to isolate total RNA from BPH samples (Life Technologies, Grand Island, NY, USA). RNA degradation and contamination were checked with 1% agarose gels. RNA purity was evaluated using a NanoPhotometer, while RNA integrity was assessed utilizing a Bioanalyzer 2100 system in conjunction with an RNA Nano 6000 Assay Kit (Agilent Technologies, Santa Clara, CA, USA).

#### 2.3.2. Library Construction, Quality Control, and Sequencing

RNA libraries were constructed using 1 µg of RNA per sample with the NEBNext Ultra RNA Library Prep Kit for Illumina, incorporating index codes for sample identification. mRNA was extracted with magnetic beads, fragmented, and converted into first-strand cDNA using random primers. The second strand was made with DNA polymerase I. Ends were blunted, adaptors added, and fragments of 250–300 bp were purified. USER Enzyme prepped the adaptor-ligated cDNA for polymerase chain reaction (PCR), carried out with high-fidelity polymerase and primers. After purification, library quality was checked using an Agilent Bioanalyzer. Samples were clustered with a cBot system and sequenced on an Illumina NovaSeq for 150 bp paired-end reads.

#### 2.3.3. PCA Analyses

We used an expression matrix containing 20,878 non-redundant genes as the input. Genes with zero expression across all samples were filtered out, resulting in a final dataset of 19,058 genes. We performed principal component analysis (PCA) and visualization using R software (version 4.0.4) and the factoextra package (version 1.0.7). The data were standardized (centered and scaled) prior to PCA to eliminate the effect of variable scales. PCA was performed using the prcomp() function. The PCA score plot (sample distribution) and variable contribution plot (gene projection) were generated using the factoextra package’s fviz_pca_ind() and fviz_pca_var() functions, respectively. In the PCA score plot, different groups were distinguished by color, and 95% confidence ellipses were added to display intergroup differences. In the variable contribution plot, a gradient color scale was used to show the contribution of each variable to the principal components.

#### 2.3.4. Clustering Analyses

We performed fuzzy clustering analysis on gene expression data using the Mfuzz package (version 2.54.0) in R software (version 4.0.4) to identify genes with similar expression patterns across different conditions or time points. The 20-cluster analysis included the following steps: Firstly, filtering missing values: genes with more than 25% missing values were removed using the filter.NA() function with the threshold set to thres = 0.25. Secondly, imputation of remaining missing values: the remaining missing values were imputed using the fill.NA() function, where the mean expression value of each gene (mode = ‘mean’) was used for imputation. Thirdly, filtering by variance: genes with a standard deviation of zero or less were removed using the filter.std() function with min.std = 0. These preprocessing steps ensured the dataset was free of significant missing values and low-variance genes, making it suitable for downstream clustering analysis.

#### 2.3.5. DEG Identification and KEGG/GO Analyses

Raw fastq data were processed with in-house perl scripts to remove poor-quality reads, adapter sequences, and poly-Ns. Clean reads were then evaluated for quality metrics like Q20, Q30, and GC content. Reference genome files were obtained from the National Center for Biotechnology Information (NCBI) database, available at: https://www.ncbi.nlm.nih.gov/datasets/genome/?taxon=108931 (Accessed 15 December 2022), indexed, and used for aligning clean reads with the Hisat2 software. Mapped reads were then assembled using StringTie, and gene-level read counts were determined with featureCounts. Transcripts Per Kilobase Million (TPM) values were calculated for each gene. Differentially expressed genes (DEGs) were identified using DESeq2, applying the following criteria: *p*-value < 0.05, false discovery rate (FDR) < 0.05, and an absolute log_2_ fold change (FC) ≥ 1. These DEGs were further analyzed for expression patterns using Mfuzz, resulting in 20 clusters, as previously described [22]. Gene Ontology (GO) and Kyoto Encyclopedia of Genes and Genomes (KEGG) analyses were performed on these clusters. GO annotations were obtained from the NCBI, available at: http://www.ncbi.nlm.nih.gov/ (Accessed 15 January 2023) and the Gene Ontology Consortium, available at: http://www.geneontology.org/ (Accessed 15 January 2023), while KEGG pathways related to BPH response were identified. Significant GO and KEGG categories were determined using Fisher’s exact tests, with *p*-value < 0.05 and FDR < 0.05.

#### 2.3.6. Heatmap Generation

The heatmap was generated using TBtools, with data derived from the log_2_ FC of DEGs across various comparisons. DEGs were identified using DESeq2 based on the following criteria: *p*-value < 0.05, FDR < 0.05, and absolute value of log_2_ FC ≥ 1. These criteria are detailed in Tables S4 and S5. All non-significant DEGs were set to zero.

### 2.4. Real-Time Quantitative PCR Determination

Total RNA was extracted from various samples of BPHs as outlined in Section 2.3.1. First-strand cDNA synthesis was performed using a PrimeScript RT Reagent Kit with gDNA Eraser (Takara, Dalian, China), following the manufacturer’s guidelines. Real-time quantitative PCR (RT-qPCR) assays for the candidate genes were conducted using 2 × M5 HiPer SYBR Premix Es Taq (Mei5bio, Beijing, China). The RT-qPCR was executed on a CFX96 Real-Time System (Bio-Rad, Philadelphia, PA, USA) with the following thermal cycling conditions: an initial denaturation at 95 °C for 5 min, followed by 40 cycles at 95 °C for 5 s, 60 °C for 30 s, and 72 °C for 30 s. Relative gene expression levels were determined using the 2^−ΔΔCt^ method, with *18S ribosomal RNA* of BPH serving as the reference gene (GenBank accession number: JN662398) [23]. The sequences of all primers used for RT-qPCR are provided in Table S1.

### 2.5. Cloning and Sequencing of the NlABCG14 Gene

The reference sequence for the *NlABCG14* gene (GenBank accession number: XM_039419461) was obtained from NCBI. Based on this reference, gene-specific primers were designed using Primer Premier 5.0 software, with details provided in Table S1. The *NlABCG14* gene was successfully amplified using specific primers, and the PCR products were subsequently ligated into the pMD-18T vector (Takara, Dalian, China). These constructs were transformed into *Escherichia coli* DH5α competent cells. The gene sequences were determined through Sanger sequencing, performed by company (HeTaiQing Biological Company, Wuhan, China).

### 2.6. Spatiotemporal Expression Patterns of NlABCG14 in Biotype Y BPH

To examine the spatio-temporal expression patterns of *NlABCG14*, the biotype Y BPH populations were reared on YHY15 rice under controlled conditions of 26 °C as previously described. For the analysis of temporal expression patterns, complete larvae of various instars, as well as adult females and males, were collected, snap-frozen using liquid nitrogen, and stored at −80 °C. Three days post-emergence of females, specific tissues and organs (leg, gut, salivary gland, fat body, and ovary) were collected and dissected using a dissector under a stereomicroscope (Leica S8APO, Wetzlar, Germany). Similarly, testes were dissected from males. Then, the samples were snap-frozen in liquid nitrogen and then stored at −80 °C for spatial expression pattern of *NlABCG14* determination. Total RNA extraction and RT-qPCR were performed as described earlier.

### 2.7. Double-Stranded RNA Synthesis, Micro-Injection, and Survival Rate Determination

Based on the cloned sequences of the *NlABCG14* gene (GenBank accession number PQ470134), dsRNA was synthesized. PCR products, specifically a 446 bp fragment of the *NlABCG14* gene, were utilized as templates for double-stranded RNA (dsRNA) synthesis, using the MEGAscript T7 transcription kit (Ambion, Austin, TX, USA). The primers used for dsRNA synthesis are detailed in Table S1. For dsRNA micro-injection, third-instar nymphs were anesthetized with carbon dioxide for 10 s, followed by the injection of approximately 20 ng of dsRNA targeting the *NlABCG14* gene (ds*NlABCG14*) using a microprocessor-controlled Nanoliter 2020 injector (World Precision Instruments, Sarasota, FL, USA). As a control, dsRNA targeting *GFP* (ds*GFP*) was also injected into third-instar nymphs. Post-injection, the revived BPHs were gently transferred to YHY15 rice plants for feeding. The survival of the BPHs was monitored and recorded every 24 h for a 16-day period. The number of live and dead BPHs was carefully observed and documented daily.

### 2.8. Measurement of BPH Weight Gain and Honeydew Excretion

The feeding capacity of BPHs on resistant rice varieties such as IR62 and AC-1613 was assessed by measuring weight gain and honeydew excretion, following established methods [24,25]. In summary, newly emerged adult female BPHs were weighed using an electronic balance (Mettler Toledo, MS105DU, Greifensee, Switzerland) and subsequently placed into pre-weighed parafilm sachets (2 × 2.5 cm) attached to the leaf sheaths of 4-week-old YHY15 rice plants. After a 48-h feeding period, the insects were carefully removed, and both the insects and honeydew collected within the sachets were weighed separately. Weight gain was assessed by comparing each insect’s weight before and after feeding, and the weight gain ratio was calculated by dividing the weight gain by the initial weight. Each assay measuring weight gain and honeydew excretion was performed with 30 replicates for both the ds*GFP* and the ds*NlABCG14* groups.

### 2.9. Statistical Analysis

Statistical analyses were performed using R software (version 4.0.4) and SPSS (version 22.0) (IBM SPSS, Somers, NY, USA) [12,26]. To determine statistically significant differences between two groups, two-sided Student’s *t*-tests were applied. For comparisons involving more than two groups, one-way ANOVA followed by Tukey’s multiple comparison tests was utilized to identify statistically significant differences.

## 3. Results

### 3.1. Transcriptional Responses of Biotype 1/Y BPH to YHY15 Rice

**Figure 1:**
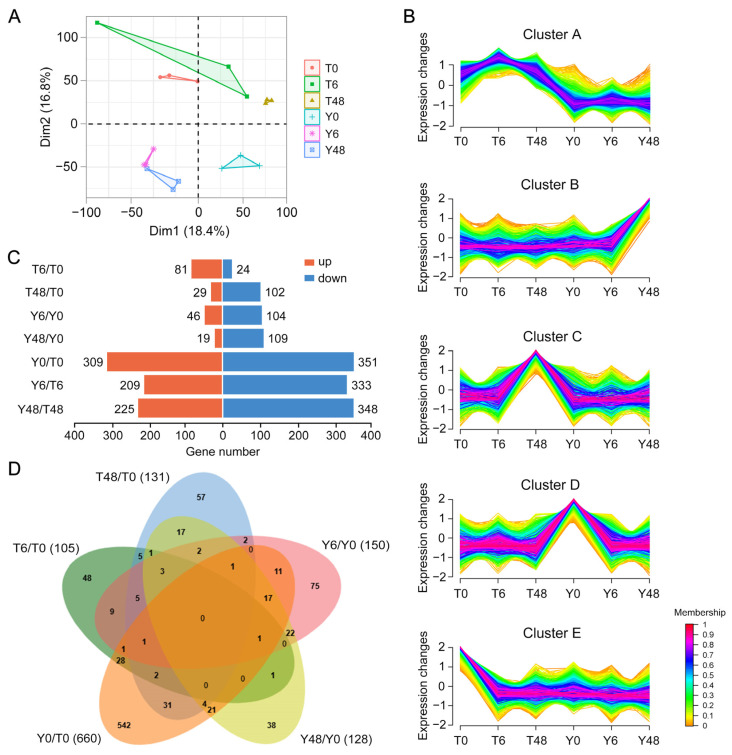
Overview of RNA-seq results. (**A**) Principal component analysis (PCA) of RNA-seq data from six sample groups. (**B**) Clustering and time-course expression of genes in biotype 1 and biotype Y BPH after feeding on YHY15 rice. The five representative distinct temporal gene expression patterns were computed with Mfuzz. The x-axis represents the six treatment groups, and the y-axis represents log_2_-transformed normalized intensity ratios for each group. Clusters A–E correspond to Clusters 3, 7, 10, 9, and 11, respectively, in Figure S1. (**C**) Number of RNAs up- or downregulated in all comparisons (log_2_ FC > 1, *p* < 0.05). (**D**) Venn diagrams of DEGs in five comparisons, including T6/T0, T48/T0, Y6/Y0, Y48/Y0, and Y0/T0. T0, non-feeding biotype 1 BPH; T6, biotype 1 BPH feeding on YHY15 seedlings for 6 h; T48, biotype 1 BPH feeding on YHY15 seedlings for 48 h; Y0, non-feeding biotype Y BPH; Y6, biotype Y BPH feeding on YHY15 seedlings for 6 h; Y48, biotype Y BPH feeding on YHY15 seedlings for 48 h.

To gain a deeper understanding of the mechanisms underlying the differential response of virulent and avirulent BPH populations to YHY15 rice, we performed RNA sequencing analyses on complete third-instar nymphs from both biotype 1 (T) and biotype Y (Y) BPHs. Principal component analysis (PCA) of RNA-seq data demonstrated a clear distinction between the samples from biotype 1 and biotype Y BPH within each respective group (Figure 1A, Table S2).

To investigate the genome-wide response of BPHs to feeding on YHY15 rice, we conducted a comprehensive transcriptome analysis covering the entire infection period. Using Mfuzz analysis, we identified 20 temporal expression clusters (Figure S1, Table S3). Subsequently, we performed KEGG and GO analyses on genes from five representative clusters to elucidate their functions (Figure 1B, Table S4). Genes in Cluster A displayed stable transcription levels in both Y6/48 and T6/48, with no change from their respective non-feeding controls (Y0 and T0). This suggests that these genes were not induced by feeding. Specifically, these genes were significantly downregulated in biotype Y BPH compared to biotype 1 BPH and were found to be enriched in cilium-related proteins, as revealed by KEGG analysis. This is indicated by relative changes against the gene’s average expression level (Figure 1B and Figure S2, Table S4). Cluster B genes displayed increased expression from 6 h to 48 h in biotype Y BPH but exhibited stable expression in the biotype 1 BPH population after feeding. These genes were associated with chromosome-associated proteins, DNA replication factors, and components of the spliceosome (Figure 1B and Figure S3, Table S4). By contrast, Cluster C genes were upregulated from 6 h to 48 h in biotype 1 BPH but remained unchanged in the biotype Y BPH population after feeding. These genes were linked to ribosomal proteins, proteasome components, translation factors, and proteins involved in signal recognition particle (SRP)-dependent co-translational targeting of proteins to membranes (Figure 1B and Figure S4A,B, Table S4). In Cluster D, gene expression remained stable in biotype 1 BPH but decreased within the first 6 h of feeding in biotype Y BPH. Conversely, in Cluster E, gene expression was stable in biotype Y BPH but decreased within the first 6 h of feeding in biotype 1 BPH (Figure 1B). Genes in Cluster D were found to be enriched in ribosome and G-protein-coupled receptor pathways according to KEGG analysis. GO analysis further indicated enrichment in structural constituents of the cuticle, SRP-dependent co-translational protein targeting to membranes, translation processes, and oxidation–reduction reactions (Figure 1B and Figure S5A,B, Table S4). Furthermore, genes in Cluster E were associated with pathways related to fatty acid biosynthesis (KEGG and GO analyses) and proteolysis (KEGG analysis) (Figure 1B and Figure S6A,B, Table S4). Overall, after feeding on YHY15 rice, biotype Y BPH displayed distinct transcriptional changes in cuticle composition, protein translocation mechanisms, and antioxidant systems. By contrast, biotype 1 BPH experienced disruptions in protein synthesis, hydrolysis, and fatty acid biosynthesis pathways. However, the precise changes in expression require further analysis.

Upon analyzing RNA expression levels throughout the feeding period, we identified 105, 131, 150, and 128 DEGs in the T6/T0, T48/T0, Y6/Y0, and Y48/Y0 comparison groups, respectively (Figure 1C, Table S5). Furthermore, by comparing the transcriptional profiles between biotype 1 BPH and biotype Y BPH, we found 660, 542, and 573 DEGs in the Y0/T0, Y6/T6, and Y48/T48 comparison groups, respectively (Figure 1C, Table S5). The expression patterns of DEGs in non-feeding BPHs as well as in avirulent and virulent BPHs at both early (6 h) and late (48 h) feeding stages are illustrated using a Venn diagram (Figure 1D, Table S6). We identified 33 overlapping DEGs between Y0/T0 and T6/T0, 39 overlapping DEGs between Y0/T0 and T48/T0, 32 overlapping DEGs between Y0/T0 and Y6/Y0, and 44 overlapping DEGs between Y0/T0 and Y48/Y0. Notably, most DEGs were specific to the Y0/T0 comparison group, suggesting that the majority of DEGs in non-feeding biotype 1 BPH and biotype Y BPH were not induced by feeding (Figure 1D, Table S6). In addition, the limited overlap of DEGs across comparisons indicated distinct response patterns in biotype 1 BPH and biotype Y BPH populations during different feeding periods (Figure 1D, Table S6). These specific responses were further analyzed in subsequent comparisons.

### 3.2. Functional Enrichment Analyses of DEGs in Responses to YHY15 Plants

**Figure 2:**
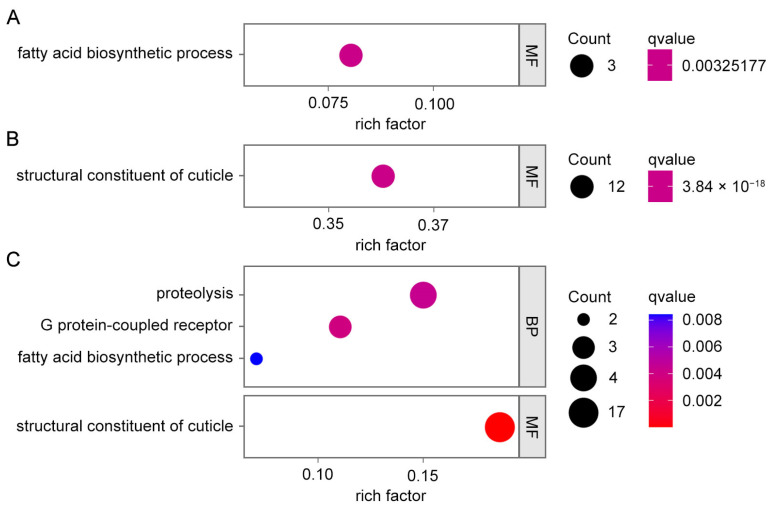
GO function analyses of DEGs specifically in T48/T0 (**A**), Y6/Y0 (**B**), and Y48/Y0 (**C**).

**Figure 3:**
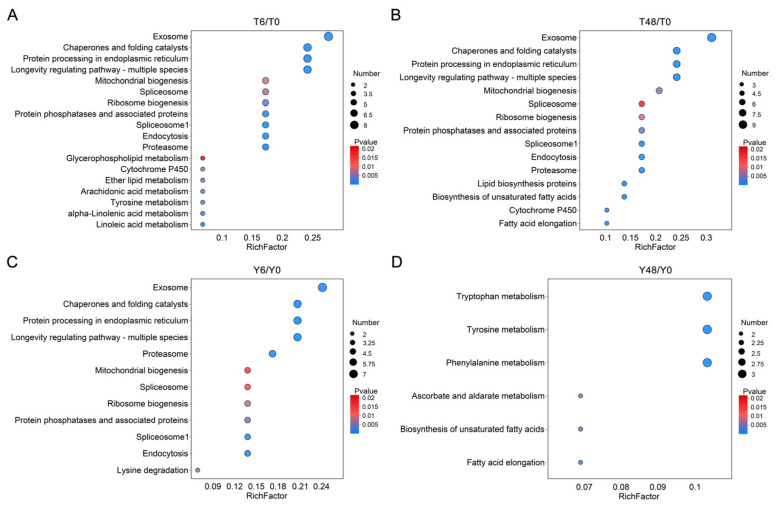
KEGG pathway enrichment analyses of DEGs specifically in T6/T0 (**A**), T48/T0 (**B**), Y6/Y0 (**C**), and Y48/Y0 (**D**).

To investigate the critical inducible pathways in BPH following feeding, we conducted comprehensive KEGG and GO analyses (Figure 2 and Figure 3, Table S7). In the T6/T0 and T48/T0 comparison groups, DEGs were significantly enriched in the pathways associated with longevity regulation, fatty acid biosynthesis, transcellular transport, cytochrome P450, mitochondrial and ribosome biogenesis, and protein processing (Figure 2A and Figure 3A,B, Table S7). Similarly, in the Y6/Y0 comparison, DEGs were enriched in the pathways associated with longevity regulation, mitochondrial and ribosome biogenesis, and protein processing, whereas the Y48/Y0 comparison showed DEG enrichment in the fatty acid biosynthesis pathway (Figure 2B,C and Figure 3C,D, Table S7). However, a marked disruption was noted in the structural components of the cuticle in both Y6/Y0 and Y48/Y0, highlighting significant response differences between biotype Y and biotype 1 BPHs (Figure 2B,C and Figure 3C,D, Table S7). The lysine degradation pathway was specifically enriched in Y6/Y0. With the extension of feeding time, several novel pathways appeared to be enriched in Y48/Y0, including the metabolism of aromatic amino acids (AAAs), such as tyrosine, tryptophan, and phenylalanine; ascorbate and aldarate metabolism; and G-protein-coupled receptor signaling (Figure 2B,C and Figure 3C,D, Table S7). In summary, feeding induced the activation of pathways related to fatty acid biosynthesis, protein processing, transcellular transport, and longevity regulation in both biotype 1 and biotype Y BPHs. By contrast, cytochrome P450 pathways were specifically enriched in biotype 1 BPH, whereas pathways associated with cuticle structure, aromatic amino acid metabolism (AAA metabolism), ascorbate and aldarate metabolism, and G-protein-coupled receptors were specifically enriched in biotype Y BPH.

### 3.3. Transcriptional Responses Related to Metabolism in Biotype 1/Y BPH to YHY15 Plants

**Figure 4:**
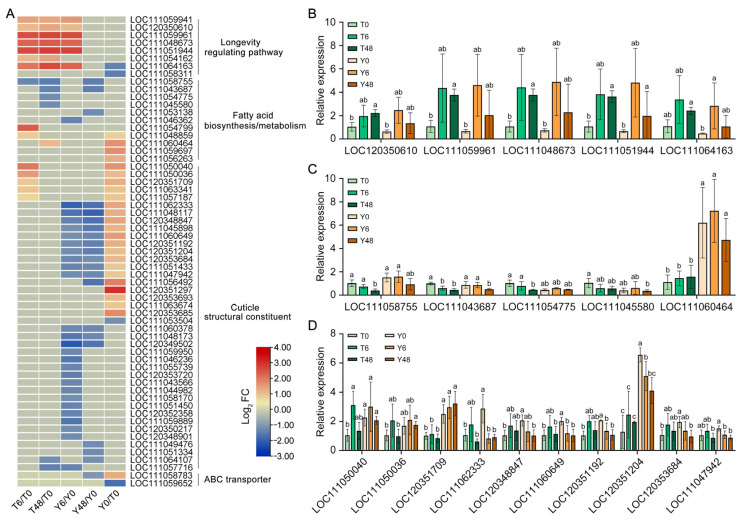
Identification of genes associated with longevity-regulating pathways, fatty acid biosynthesis/metabolism, cuticle structural constituents, and ABC transporters in the BPH transcriptome. (**A**) Heatmap of average log_2_ FC of DEGs in five comparisons associated with specific pathways in RNA-seq. All non-significant DEGs were set to zero. The five comparisons, including T6/T0, T48/T0, Y6/Y0, Y48/Y0, and Y0/T0, are as follows: T0, non-feeding biotype 1 BPH; T6, biotype 1 BPH feeding on YHY15 seedlings for 6 h; T48, biotype 1 BPH feeding on YHY15 seedlings for 48 h; Y0, non-feeding biotype Y BPH; Y6, biotype Y BPH feeding on YHY15 seedlings for 6 h; Y48, biotype Y BPH feeding on YHY15 seedlings for 48 h. (**B**) RT-qPCR analysis of DEGs associated with the longevity-regulating pathway. (**C**) RT-qPCR analysis of DEGs associated with fatty acid biosynthesis/metabolism. (**D**) RT-qPCR analysis of DEGs associated with cuticle structural constituent. Moreover, 18S ribosomal RNA was used as the reference gene to normalize gene expression. Bars are the mean ± SEM (standard error of mean) from three independent experiments. The data were analyzed by one-way ANOVA and Tukey’s multiple comparison tests. Data are presented as means ± SEM from three biological replicates. Bars labeled with different lowercase letters indicate statistically significant differences among groups, as determined by ANOVA followed by Tukey’s Honestly Significant Difference test (*p* < 0.05).

To further investigate changes in the expression of genes related to key metabolic pathways, such as longevity regulation, fatty acid biosynthesis/metabolism, and cuticle structural constituents, we visualized these changes using a heatmap and validated the RNA-seq findings through RT-qPCR (Figure 4, Table S8). We compared the expression trends in BPHs after feeding (T6/T0, T48/T0, Y6/Y0, and Y48/Y0) and in the two non-feeding BPH populations (Y0/T0; Figure 4A). Overall, most DEGs associated with the longevity regulation pathway were upregulated in both BPH biotypes after feeding on YHY15 rice, with a particularly strong and sustained increase observed in biotype 1 BPH within 48 h (Figure 4A). These results were corroborated by those of RT-qPCR (Figure 4B). Further analyses revealed that many of these genes encode heat shock proteins (HSPs), which are known stress-related proteins (Table S8), suggesting a pronounced stress response in biotype 1 BPH following feeding on YHY15 rice.

Analysis of the expression of DEGs involved in fatty acid biosynthesis/metabolism revealed that feeding induced the downregulation of six DEGs in either biotype 1 or biotype Y BPH but caused upregulation of three DEGs specifically in biotype 1 BPH (Figure 4A,C). Furthermore, in the two non-feeding BPH populations, four genes showed higher transcriptional levels in biotype Y BPH than in biotype 1 BPH (Figure 4A,C). These observations suggest different basal levels and distinct responses in the fatty acid pathway between the two BPH biotypes following feeding on YHY15 rice.

Genes associated with cuticle structural constituents exhibited significant expression differences between biotype 1 and biotype Y BPH populations. In the two non-feeding BPH biotypes, 20 genes exhibited higher transcriptional levels in biotype Y BPH than in biotype 1 BPH. However, feeding on YHY15 rice induced upregulation of certain genes in biotype 1 BPH and downregulation of some DEGs in biotype Y BPH (Figure 4A,D).

Moreover, we observed a significant increase in the expression of genes involved in phenylpropanoid biosynthesis in YHY15 rice seedlings infested with BPHs, particularly biotype Y BPH. This upregulation may trigger metabolic responses in feeding BPHs [12]. ABC transporters, which are essential for managing nutrient transport, development, osmoregulation, detoxification, and immune defense in insects, were found to exhibit different baseline transcriptional levels and response patterns in the two BPH biotypes (Figure 4A). The differential transcriptional responses of ABC transporter genes in biotype 1 and biotype Y BPH populations suggest that these genes play crucial roles in the responses of BPHs to resistant rice. Notably, *NlABCG14* (LOC111058783 or XM_039419461) exhibited higher basal transcriptional levels in biotype Y BPH compared to biotype 1 BPH. Conversely, *NlABCG15* (LOC111059652) displayed the opposite trend. This suggests that *NlABCG14* may play a potential role in the feeding response to resistant rice.

### 3.4. Effect of NlABCG14 Gene Silencing on Honeydew Production in Biotype Y BPH

**Figure 5:**
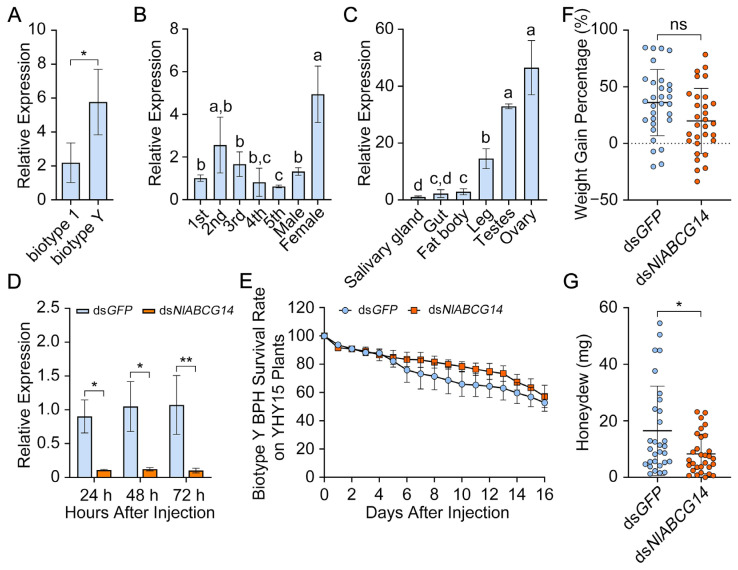
Spatiotemporal expression pattern of *NlABCG14* in biotype Y BPH and the impact of its silencing on honeydew production reduction when feeding on YHY15 rice. (**A**) RT-qPCR analysis of *NlABCG14* mRNA levels in entire third-instar nymphs of biotype 1 or biotype Y BPH, respectively. (**B**) Temporal expression pattern of *NlABCG14* in biotype Y BPH, spanning from nymphs (first to fifth instar) to male and female adults (3 days post-emergence). (**C**) Spatial expression pattern of *NlABCG14* across various tissues, including salivary gland (Sg), gut (Gut), fat body (Fb), leg (Leg), testes (Te), and ovary (Ov). (**D**) Relative expression levels of the *NlABCG14* gene in third-instar nymphs after dsRNA injection, with ds*GFP* serving as a control. (**E**) Impact of ds*NlABCG14* interference on the survival of biotype Y BPH when feeding on YHY15 rice. (**F**) Impact of ds*NlABCG14* interference on the weight gain percentage of biotype Y BPH on YHY15 rice. (**G**) Impact of ds*NlABCG14* interference on the honeydew production of biotype Y BPH on YHY15 rice. Data were calculated from three independent experiments, each of which included at least 30 BPHs. Gene expression was normalized using *18S ribosomal RNA* of BPH as the reference gene. Asterisks denote statistically significant differences in honeydew production between ds*NlABCG14* and ds*GFP* groups (Statistical analysis was performed as follows: “ns” indicates no significant difference, while * *p* < 0.05, ** *p* < 0.01 denote significant differences based on a two-tailed Student’s *t*-test. Different letters indicate statistically significant differences determined by one-way ANOVA followed by the LSD post hoc test, *p* < 0.05).

To further validate the transcriptional patterns of *NlABCG14*, RT-qPCR was performed using third-instar nymphs of biotype 1 and biotype Y BPH. The results showed that the expression level of *NlABCG14* was significantly higher in biotype Y BPH compared to biotype 1, consistent with the transcriptome data (Figure 5A). To verify the function of *NlABCG14*, its spatiotemporal expression pattern was initially assessed through RT-qPCR. Subsequently, dsRNA targeting *NlABCG14* was injected into third-instar nymphs of BPH, and their survival rates and growth indices were monitored. This experimental approach facilitated the determination of the specific role of *NlABCG14* in response to the resistant rice variety YHY15. First, the spatiotemporal expression patterns indicated that *NlABCG14* was consistently expressed across all developmental stages and the examined tissues, with markedly higher expression observed in female adults and ovaries (Figure 5B,C). These findings suggest that *NlABCG14* plays a broad functional role throughout the lifespan of BPH. To study the effect of *NlABCG14* knockdown on biotype Y BPHs, we cloned and sequenced the gene to synthesize dsRNA (ds*NlABCG14*), injected it into third-instar nymphs, and used RT-qPCR to assess knockdown efficiency. The results of RT-qPCR revealed significantly decreased *NlABCG14* expression levels at 24, 48, and 72 h post-injection compared with the control group (injected with ds*GFP*) (Figure 5D), confirming successful gene silencing. However, the survival rate of BPHs injected with ds*NlABCG14* on YHY15 rice did not differ significantly from that of the control group injected with ds*GFP* (Figure 5E). In addition, no significant changes were observed in weight gain percentage, a common growth index, suggesting that *NlABCG14* does not significantly affect the survival and growth of biotype Y BPH on YHY15 rice (Figure 5E,F). Despite this, it is worth noting that the honeydew production significantly decreased on YHY15 rice when *NlABCG14* was silenced in biotype Y BPH (Figure 5G). This decrease in honeydew production, a reflection of the feeding response of BPHs [27,28,29,30,31,32], suggests that *NlABCG14* plays a role in the feeding response of biotype Y BPH to this resistant rice variety.

## 4. Discussion

The coevolutionary arms race between insects and plants has led to the emergence of virulent insect variations and resistant plant traits [33]. Virulent insect variations include genetic adaptations that enable them to bypass plant defenses, whereas resistant plants develop traits to mitigate or prevent damage induced by herbivorous insects [16,34,35,36]. In this study, a short-term feeding experiment with both avirulent (biotype 1 BPH) and virulent (biotype Y BPH) on resistant rice varieties was conducted, revealing significant transcriptional reprogramming. This response is consistent with patterns observed in other studies [19,37,38,39]. Understanding this dynamic interaction is vital for sustainable agriculture and developing effective pest management strategies. The interaction of avirulent and virulent BPH populations with resistant YHY15 rice serves as an ideal model to study the mechanisms underlying virulence in BPHs. Our previous study combined miRNA and mRNA sequencing to identify defense-related metabolic pathways in YHY15 rice that are activated in response to feeding in biotype Y BPH populations [12]. Building on this foundation, the current study examined the different response mechanisms in both biotype 1 and biotype Y BPH when exposed to the defensive system in YHY15 rice. Insights from this research are critical for developing targeted pest management strategies that can effectively shield rice crops from the evolving threats posed by BPHs.

BPH feeding also induces the activation of the phenylpropanoid synthesis pathway—a critical precursor for various defensive metabolites such as flavonoids, phenolic acids, lignin, tannins, phenolic compounds, and stilbenes—in YHY15 rice seedlings [12,14]. The metabolites derived from phenylpropanoids enhance plant defense against herbivores by reducing palatability, inhibiting digestion, disrupting feeding behavior, and exerting direct toxic effects on herbivores [40,41,42,43]. However, insects have evolved response strategies that enable them to ingest, metabolize, and eliminate toxic substances encountered in their environment or ingested from plant sources. The present study identified *NlABCG14*, an ABC transporter, as playing a role in the feeding amount of biotype Y BPH on YHY15 rice. Silencing *NlABCG14* led to decreased honeydew production. We hypothesize that the translocation function of *NlABCG14* aids BPHs in ingesting and digesting defensive compounds from resistant rice. This hypothesis is supported by the broad expression of *NlABCG14* in various digestive organs, such as the salivary glands, gut, and fat body, throughout the insect’s lifespan. Similarly, numerous studies have highlighted the pivotal roles of other ABC transporters in insect resistance to pesticides or insecticidal proteins [44,45,46]. This study extends previous findings, underscoring their function in response to resistant plants, suggesting that the ABC transporters in BPH play roles in their feeding response on resistant rice.

Another gene family, known as the heat shock protein (HSP) gene family, is part of the longevity regulatory pathway. The HSP gene family plays crucial roles in ambient temperature, pesticides, and diapause in insects [47]. For instance, the *NlHSP90* gene plays an important role in the heat tolerance of BPHs [48]. In addition to the response to temperature, *NlHSP70* in BPHs is essential for resistance to insecticide exposure [49]. Moreover, the 70 kDa heat-shock protein cognate 3 (NlHSC70-3) of BPHs may function as an effector to facilitate pest survival on rice by manipulating plant physiological processes [50]. However, it is not clear whether the HSP gene mediates the adaptation of the BPH to resistant plants. In this study, we found that the expression of *HSPs* in both biotype 1 and biotype Y BPHs was upregulated after they fed on YHY15 rice, suggesting their potential roles in mediating the adaptation of BPHs to resistant plants.

In addition, we observed distinct responses in the fatty acid-related pathways between biotype 1 and biotype Y BPH populations. Fatty acid biosynthesis and metabolism are critical processes in insects, playing key roles in energy production, membrane structure, signaling pathways, and energy storage. The observed differences in fatty acid biosynthesis and metabolism between the two BPH biotypes may be linked to their varying feeding efficiencies. Biotype Y BPH, which consumed more YHY15 rice than biotype 1 BPH—as indicated by their weight gain and honeydew excretion data—could significantly regulate their growth, development, reproduction, and adaptation to environmental changes [12].

Many genes related to cuticle structure exhibited higher transcriptional levels in non-feeding biotype Y BPH than in biotype 1 BPH. However, feeding on YHY15 rice induced downregulation of most of these genes in biotype Y BPH and upregulation of some genes in biotype 1 BPH. The insect cuticle is a critical multifunctional structure for various physiological roles and survival mechanisms, such as protection, structural support, water loss prevention, gas exchange, sensory functions, pigmentation and camouflage, molting, growth, and development stages, including nymph to adult transitions and egg or embryo development [28]. Dynamic changes in the transcription profiles of cuticle formation-related genes may positively impact their adaptation to resistant rice varieties. For example, because resistant plants emit chemical defenses to deter herbivores, the insect cuticle acts as a barrier that limits the absorption of these chemicals into the body. Continuous feeding on resistant plants may drive evolutionary adaptations in the cuticle, such as increased thickness or impermeability, enhancing the insects’ ability to withstand plant defenses. This adaptation could improve their survival and reproductive success on resistant plant species. Additionally, melanin derived from AAA metabolism contributes to the cuticle’s hardening, pigmentation, wound healing, and immune responses. Disruption in the transcription of this pathway might also affect these functions [51,52]. However, this hypothesis warrants further investigation.

## 5. Conclusions

In conclusion, our findings highlight the role of the metabolic system, particularly the ABC transporter, *NlABCG14*, present in biotype Y BPH in their feeding response to YHY15 and resistant rice varieties. The ingestion and digestive responses are also potentially attributed to the pathways related to fatty acids and cuticle development. These discoveries enhance our understanding of the complex molecular dynamics in rice–BPH interactions and provide a foundation for further exploration of specific genes, pathways, and regulatory elements associated with the adaptation of various BPH populations to resistant rice varieties.

## Data Availability

All raw RNA sequencing data generated in this study has been deposited in the NCBI SRA database under the BioProject number PRJNA1149491.

## Supplementary Materials

The following supporting information can be downloaded at: https://www.mdpi.com/article/10.3390/insects15120992/s1. Figure S1: Clustering and time-course expression of genes after BPH feeding; Figure S2: KEGG pathway enrichment analyses of cluster A; Figure S3: KEGG pathway enrichment analyses of cluster B; Figure S4: KEGG pathway enrichment analyses (A) and GO function analyses (B) of genes in cluster C; Figure S5: KEGG pathway enrichment analyses (A) and GO function analyses (B) of genes in cluster D; Figure S6: KEGG pathway enrichment analyses (A) and GO function analyses (B) of genes in cluster E; Table S1: Sequence of primers in this study; Table S2: Gene expression lists of all samples; Table S3: Gene expression lists of the 20 clusters; Table S4: Gene expression lists and KEGG/GO analysis of the representative five clusters; Table S5: DEGs in all comparisons; Table S6: DEGs overlapped in multiple comparisons; Table S7: GO function enrichment and KEGG pathway analyses of DEGs in biotype 1/Y BPH; Table S8: The gene list shown in the heatmap.

## References

[1] Disclaim Gallagher K.D., Kenmore P.E., Sogawa K., Denno R.F., Perfect T.J. (1994). Judicial use of insecticides deter planthopper outbreaks and extend the life of resistant varieties in southeast Asian rice. Planthoppers: Their Ecology and Management.

[2] Rivera C., Ou S., Iida T. (1966). Grassy stunt disease of rice and its transmission by the planthopper *Nilaparvata lugens* Stål. Plant Dis. Rep..

[3] Ling K.C., Tiongco E.R., Aguiero V.M. (1978). Rice ragged stunt, a new virus disease. Plant Dis. Rep..

[4] Cheng X.Y., Zhu L.L., He G.C. (2013). Towards understanding of molecular interactions between rice and the brown planthopper. Mol. Plant..

[5] Wang H.Y., Shi S.J., Hua W. (2023). Advances of herbivore-secreted elicitors and effectors in plant-insect interactions. Front. Plant Sci..

[6] Yang H.Y., You A.Q., Yang Z.F., Zhang F.T., He R.F., Zhu L.L., He G.C. (2004). High resolution genetic mapping at the *BPH15* locus for brown planthopper resistance in rice (*Oryza sativa* L.). Theor. Appl. Genet..

[7] Lv W.T., Du B., Shangguan X.X., Zhao Y., Pan Y.F., Zhu L.L., He Y.Q., He G.C. (2014). BAC and RNA sequencing reveal the brown planthopper resistance gene *BPH15* in a recombination cold spot that mediates a unique defense mechanism. BMC Genom..

[8] Peng L., Zhao Y., Wang H.Y., Zhang J.J., Song C.P., Shangguan X.X., Zhu L.L., He G.C. (2016). Comparative metabolomics of the interaction between rice and the brown planthopper. Metabolomics.

[9] Herrmann K.M. (1995). The shikimate pathway as an entry to aromatic secondary metabolism. Plant Physiol..

[10] Weaver L.M., Herrmann K.M. (1997). Dynamics of the shikimate pathway in plants. Trends Plant Sci..

[11] Maeda H., Dudareva N. (2012). The shikimate pathway and aromatic amino acid biosynthesis in plants. Annu. Rev. Plant Biol..

[12] Yu B., Geng M.J., Xue Y., Yu Q.Q., Lu B.J., Liu M., Shao Y.H., Li C.X., Xu J.G., Li J.T. (2024). Combined miRNA and mRNA sequencing reveals the defensive strategies of resistant YHY15 rice against differentially virulent brown planthoppers. Front. Plant Sci..

[13] Hao P.Y., Liu C.X., Wang Y.Y., Chen R.Z., Tang M., Du B., Zhu L., He G.C. (2008). Herbivore induced callose deposition on the sieve plates of rice: An important mechanism for host resistance. Plant Physiol..

[14] Dong N.Q., Lin H.X. (2021). Contribution of phenylpropanoid metabolism to plant development and plant–environment interactions. J. Integr. Plant Biol..

[15] Alam S.N., Cohen M.B. (1998). Durability of brown planthopper, *Nilaparvata lugens*, resistance in rice variety IR64 in greenhouse selection studies. Entomol. Exp. Appl..

[16] Jing S.L., Liu B.F., Peng L., Peng X.X., Zhu L.L., Fu Q., He G.C. (2011). Development and use of EST-SSR markers for assessing genetic diversity in the brown planthopper (*Nilaparvata lugens* Stål). Bull. Entomol. Res..

[17] Zheng Y., He J.C., Wan P.J., Lai F.X., Sun Y.Q., Lin J.J., Fu Q. (2016). Virulence characteristics of *Nilaparvata lugens* (Stål) reared on resistant rice variety IR56. Chin. J. Rice Sci..

[18] Ji R., Yu H.X., Fu Q., Chen H.D., Ye W.F., Li S.H., Lou Y.G. (2013). Comparative transcriptome analysis of salivary glands of two populations of rice brown planthopper, *Nilaparvata lugens*, that differ in virulence. PLoS ONE.

[19] Wan P.J., Zhou R.N., Nanda S., He J.C., Yuan S.Y., Wang W.X., Lai F.X., Fu Q. (2019). Phenotypic and transcriptomic responses of two *Nilaparvata lugens* populations to the Mudgo rice containing *Bph1*. Sci. Rep..

[20] Guan W., Shan J.H., Gao M.Y., Guo J.P., Wu D., Zhang Q., Wang J., Chen R.Z., Du B., Zhu L.L. (2022). Bulked segregant RNA sequencing revealed difference between virulent and avirulent brown planthoppers. Front. Plant Sci..

[21] Zha W.J., Zhou L., Li S.H., Liu K., Yang G.C., Chen Z.J., Liu K., Xu H.S., Li P.D., Hussain S. (2016). Characterization and comparative profiling of the small RNA transcriptomes in the Hemipteran insect *Nilaparvata lugens*. Gene.

[22] Kumar L., Futschik M. (2007). Mfuzz: A software package for soft clustering of microarray data. Bioinformation.

[23] Peng L.Y., Dai Z.W., Yang R.R., Zhu Z., Wang W., Zhou X., Bao Y.Y. (2020). NADPH oxidase 5 is essential for molting and oviposition in a rice planthopper *Nilaparvata lugens*. Insects.

[24] Horgan F.G., Cruz A.P., Arriza A., Ferrater J.B., Bernal C.C. (2021). Adaptation by the brown planthopper to resistant rice: A test of female-derived virulence and the role of yeast-like symbionts. Insects.

[25] Shi S.J., Wang H.Y., Nie L.Y., Tan D., Zhou C., Zhan Q., Li Y., Du B., Guo J.P., Huang J. (2021). *Bph30* confers resistance to brown planthopper by fortifying sclerenchyma in rice leaf sheaths. Mol. Plant.

[26] Ritchie M.E., Phipson B., Wu D., Hu Y., Law C.W., Shi W., Smyth G.K. (2015). *limma* powers differential expression analyses for RNA-sequencing and microarray studies. Nucleic Acids Res..

[27] Backus E.A., Serrano M.S., Ranger C.M. (2005). Mechanisms of Hoppernurn: An overview of insect taxonomy, behavior, and physiology. Annu. Rev. Entomol..

[28] AB Ghaffar M.B., Pritchard J., Ford-Lloyd B. (2011). Brown planthopper (*N. lugens* Stål ) feeding behaviour on rice germplasm as an indicator of resistance. PLoS ONE.

[29] Khan Z.R., Saxenn R.C. (1988). Probing behavior of three biotypes of *Nilaparvata lugens* (Homoptera: Delphacidae) on different resistant and susceptible rice varieties. J. Econ. Entomol..

[30] Kimmins E.M. (1989). Electrical penetration graphs from *Nilaparvata lugens* on resistant and susceptible rice varieties. Entomol. Exp. Appl..

[31] Cook A.G., Denno R.F. (1994). Planthopper/Plant interactions: Feeding behavior, plant nutrition, plant defense, and host plant specialization. Planthoppers: Their Ecology and Management.

[32] Cao T.T., Backus E.A., Lou Y.G., Cheng J.A. (2013). Feeding-induced interactions between *Nilaparvata lugens* and *Laodelphax striatellus* (Hemiptera: Delphacidae): Effects on feeding behavior and honeydew excretion. Environ. Entomol..

[33] Erb M., Reymond P. (2019). Molecular interactions between plants and insect herbivores. Annu. Rev. Plant Biol..

[34] Howe G.A., Jander G. (2008). Plant immunity to insect herbivores. Annu. Rev. Plant Biol..

[35] Schuman M.C., Baldwin I.T. (2015). The layers of plant responses to insect herbivores. Annu. Rev. Entomol..

[36] Zu P.J., Boege K., Del-Val E., Schuman M.C., Stevenson P.C., Zaldivar-Riverón A., Saavedra S. (2020). Information arms race explains plant-herbivore chemical communication in ecological communities. Science.

[37] Pym A., Singh K.S., Nordgren Å., Davies T.G.E., Zimmer C.T., Elias J., Slater R., Bass C. (2019). Host plant adaptation in the polyphagous whitefly, *Trialeurodes vaporariorum*, is associated with transcriptional plasticity and altered sensitivity to insecticides. BMC Genom..

[38] Rout P., Ravindranath N., Gaikwad D., Nanda S. (2023). Unveiling *Nilaparvata lugens* Stål genes defining compatible and incompatible interactions with rice through transcriptome analysis and gene silencing. Curr. Issues Mol. Biol..

[39] Wybouw N., Zhurov V., Martel C., Bruinsma K.A., Hendrickx F., Grbić V., Van Leeuwen T. (2015). Adaptation of a polyphagous herbivore to a novel host plant extensively shapes the transcriptome of herbivore and host. Mol. Ecol..

[40] Fraser C.M., Chapple C. (2011). The phenylpropanoid pathway in Arabidopsis. Am. Soc. Plant Biol..

[41] Gaquerel E., Gulati J., Baldwin I.T. (2014). Revealing insect herbivory-induced phenolamide metabolism: From single genes to metabolic network plasticity analysis. Plant J..

[42] Singh S., Kaur I., Kariyat R. (2021). The multifunctional roles of polyphenols in plant-herbivore interactions. Int. J. Mol. Sci..

[43] Grover S., Shinde S., Puri H., Palmer N., Sarath G., Sattler S.E., Louis J. (2022). Dynamic regulation of phenylpropanoid pathway metabolites in modulating sorghum defense against fall armyworm. Front. Plant Sci..

[44] Heckel D.G. (2021). The essential and enigmatic role of ABC transporters in Bt resistance of noctuids and other insect pests of agriculture. Insects.

[45] Xu H.Q., Ma M., Ma Y.P., Zhang S.Y., Li W.J., Wei D., Wang J.J. (2021). Identification and expression characterization of ATP-binding cassette (ABC) transporter genes in melon fly. Insects.

[46] Amezian D., Nauen R., Leeuwen T.V. (2024). The role of ATP-binding cassette transporters in arthropod pesticide toxicity and resistance. Curr. Opin. Insect Sci..

[47] King A.M., MacRae T.M. (2015). Insect heat shock proteins during stress and diapause. Annu. Rev. Entomol..

[48] Lu K., Chen X., Liu W.T., Zhou Q. (2016). Identification of a heat shock protein 90 gene involved in resistance to temperature stress in two wing-morphs of *Nilaparvata lugens* (Stål). Comp. Biochem. Phys. A.

[49] Lu K., Chen X., Liu W.T., Zhang Z.C., Wang Y., You K.K., Li Y., Zhang R.B., Zhou Q. (2017). Characterization of heat shock protein 70 transcript from *Nilaparvata lugens* (Stål): Its response to temperature and insecticide stresses. Pestic. Biochem. Phys..

[50] Yang H.H., Zhang X.Y., Li H.J., Ye Y.Y., Li Z.P., Han X., Hu Y.R., Zhang C.X., Jiang Y.J. (2022). Heat shock 70 kDa protein cognate 3 of brown planthopper is required for survival and suppresses immune response in plants. Insects.

[51] Parthasarathy A., Cross P.J., Dobson R.J., Adams L.E., Savka M.A., Hudson A.O. (2018). A three-ring circus: Metabolism of the three proteogenic aromatic amino acids and their role in the health of plants and animals. Front. Mol. Bio. Sci..

[52] Brunet P.C.J. (1980). The metabolism of the aromatic amino acids concerned in the cross-linking of insect cuticle. Insect Biochem..

