# Supplementary figures and images for: Comparative Transcriptome Analysis Highlights the Role of NlABCG14 in the Honeydew Production of Virulent Brown Planthoppers (Nilaparvata lugens Stål) to Resistant Rice Variety
Source: Insects. 2024 Dec 15;15(12):992. doi: 10.3390/insects15120992 (PMC11676699; doi:10.3390/insects15120992)

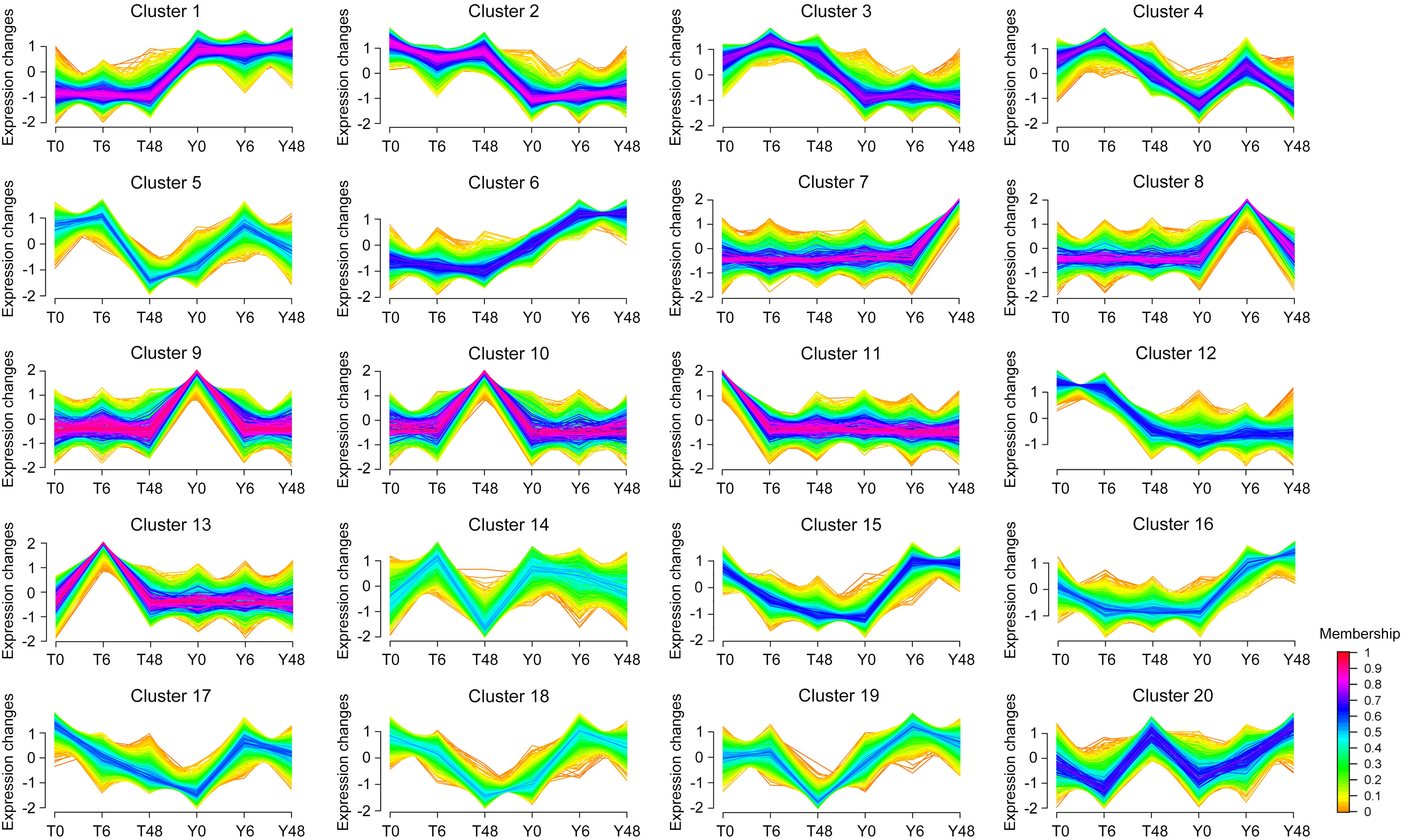

Supplement: Supplementary file 1 [file insects-15-00992-s001.zip › Figure S1 Clustering and time-course expression of genes after BPH feeding.jpg]

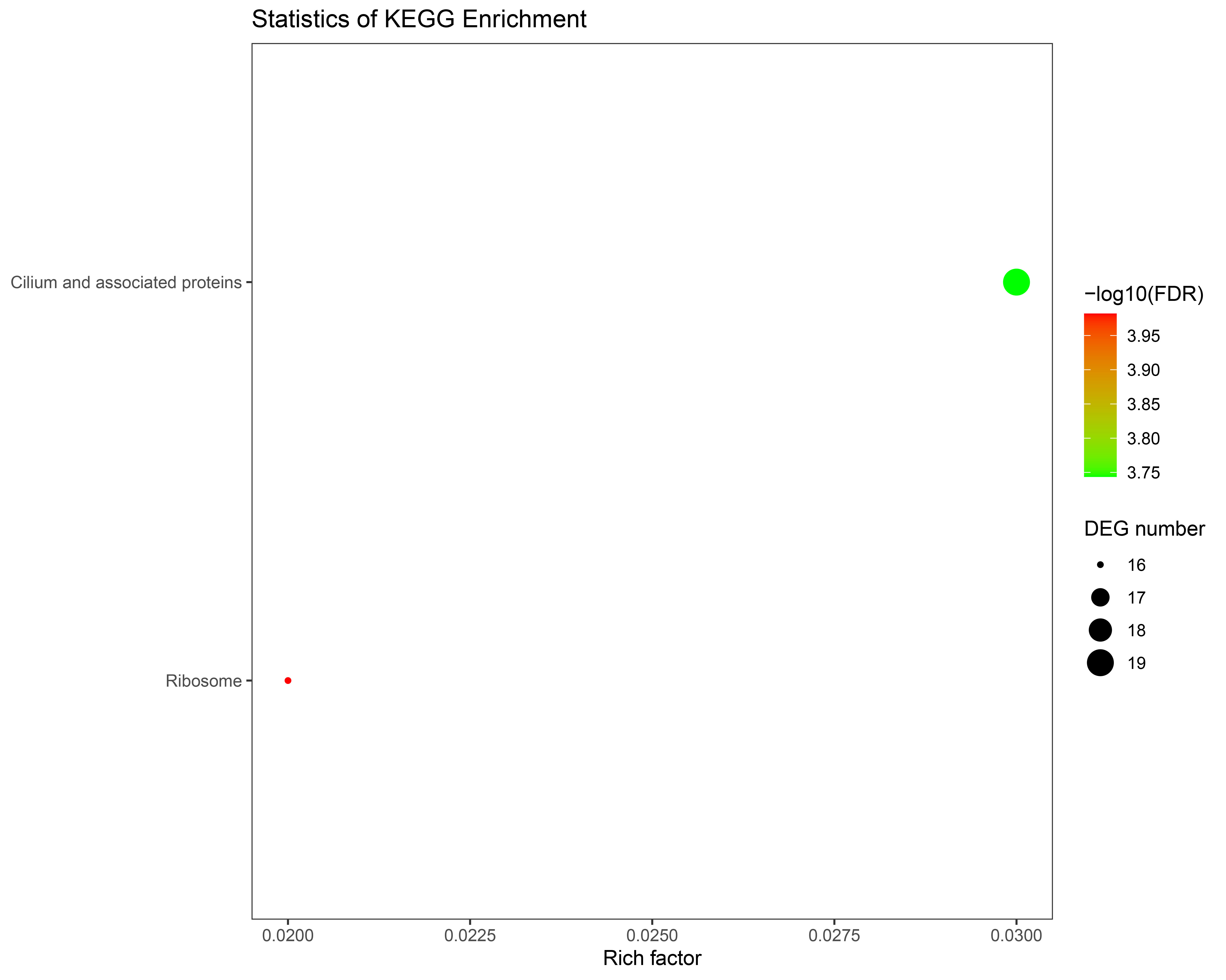

Supplement: Supplementary file 1 [file insects-15-00992-s001.zip › Figure S2 KEGG pathway enrichment analyses of cluster A.jpg]

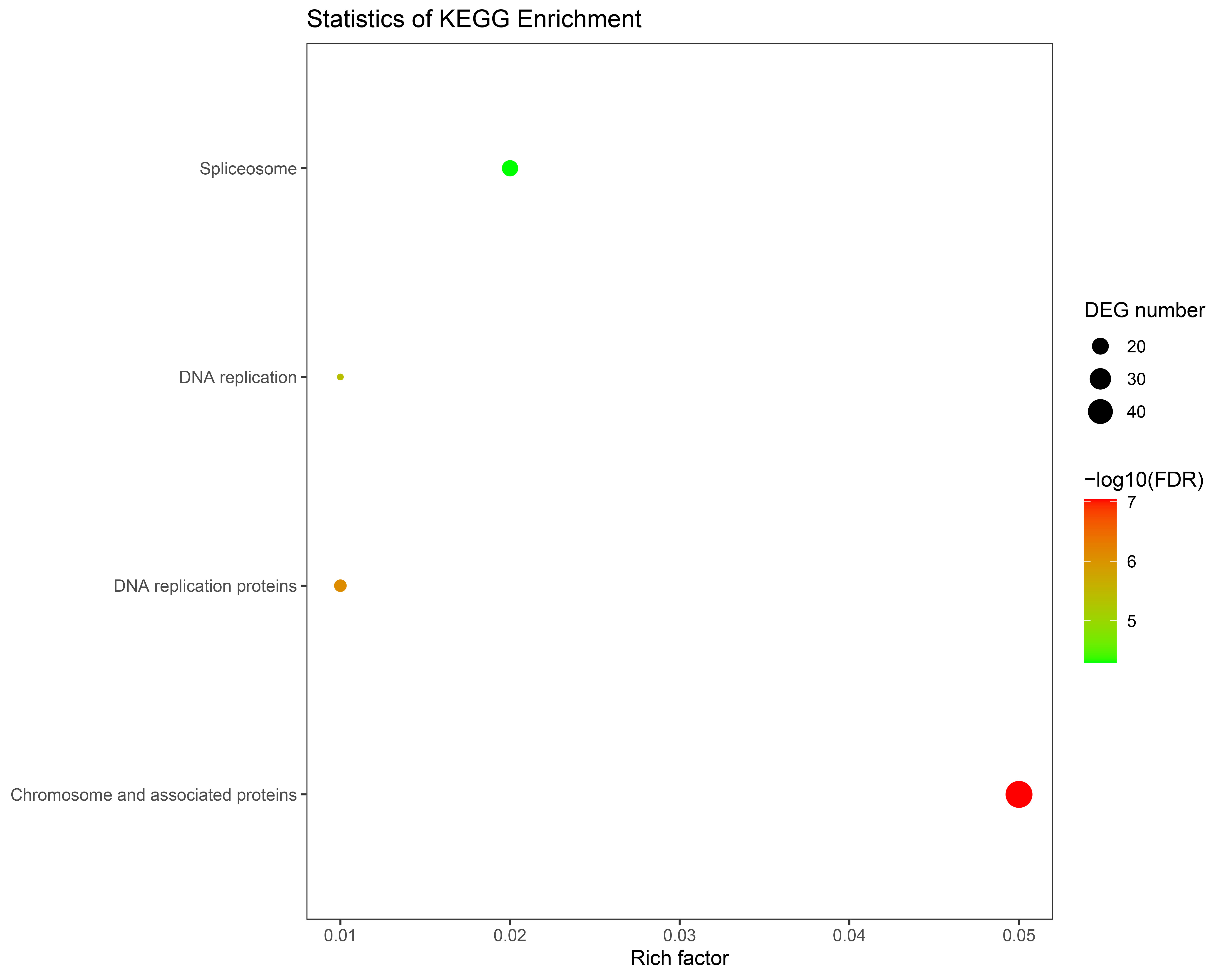

Supplement: Supplementary file 1 [file insects-15-00992-s001.zip › Figure S3 KEGG pathway enrichment analyses of cluster B.jpg]

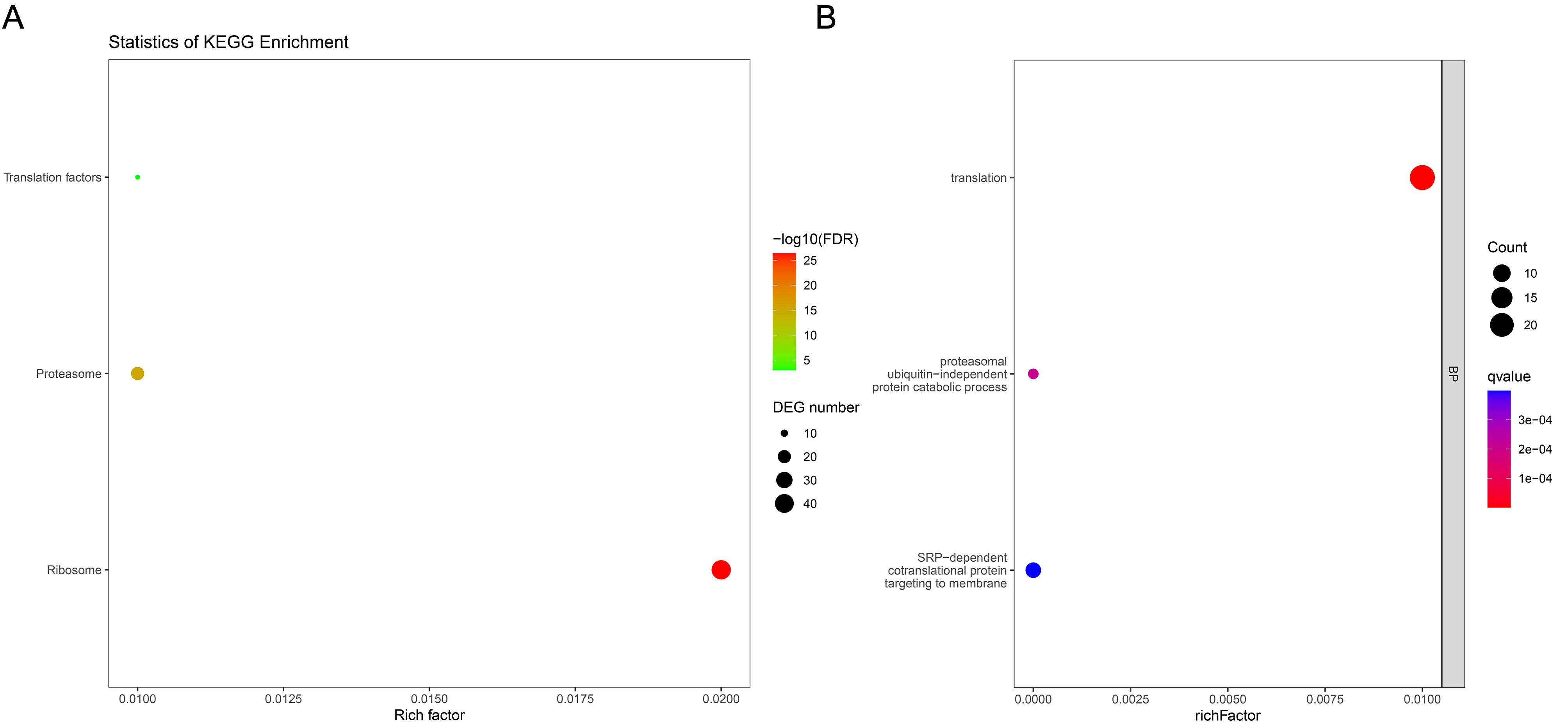

Supplement: Supplementary file 1 [file insects-15-00992-s001.zip › Figure S4 KEGG pathway enrichment analyses (A) and GO function analyses (B) of genes in cluster C.tif]

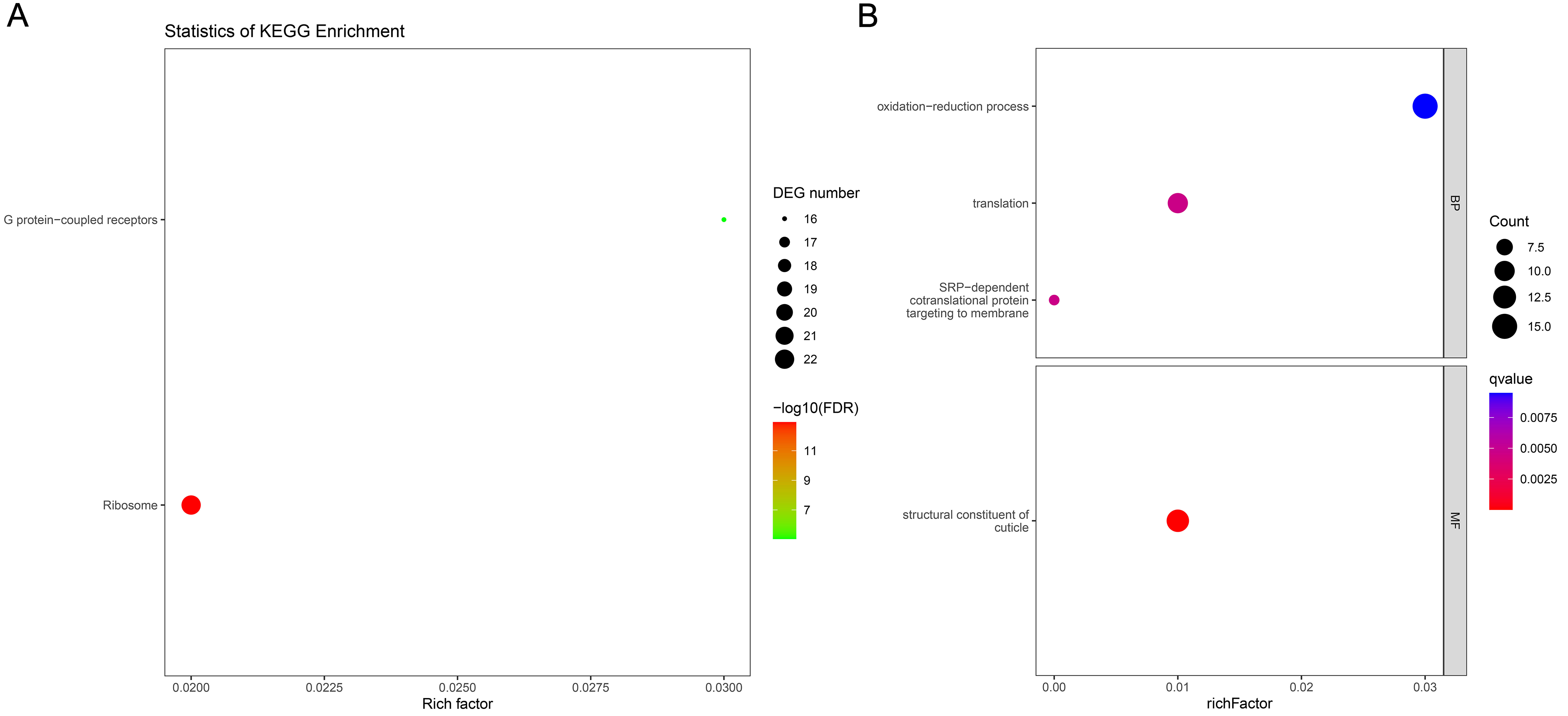

Supplement: Supplementary file 1 [file insects-15-00992-s001.zip › Figure S5 KEGG pathway enrichment analyses (A) and GO function analyses (B) of genes in cluster D.tif]

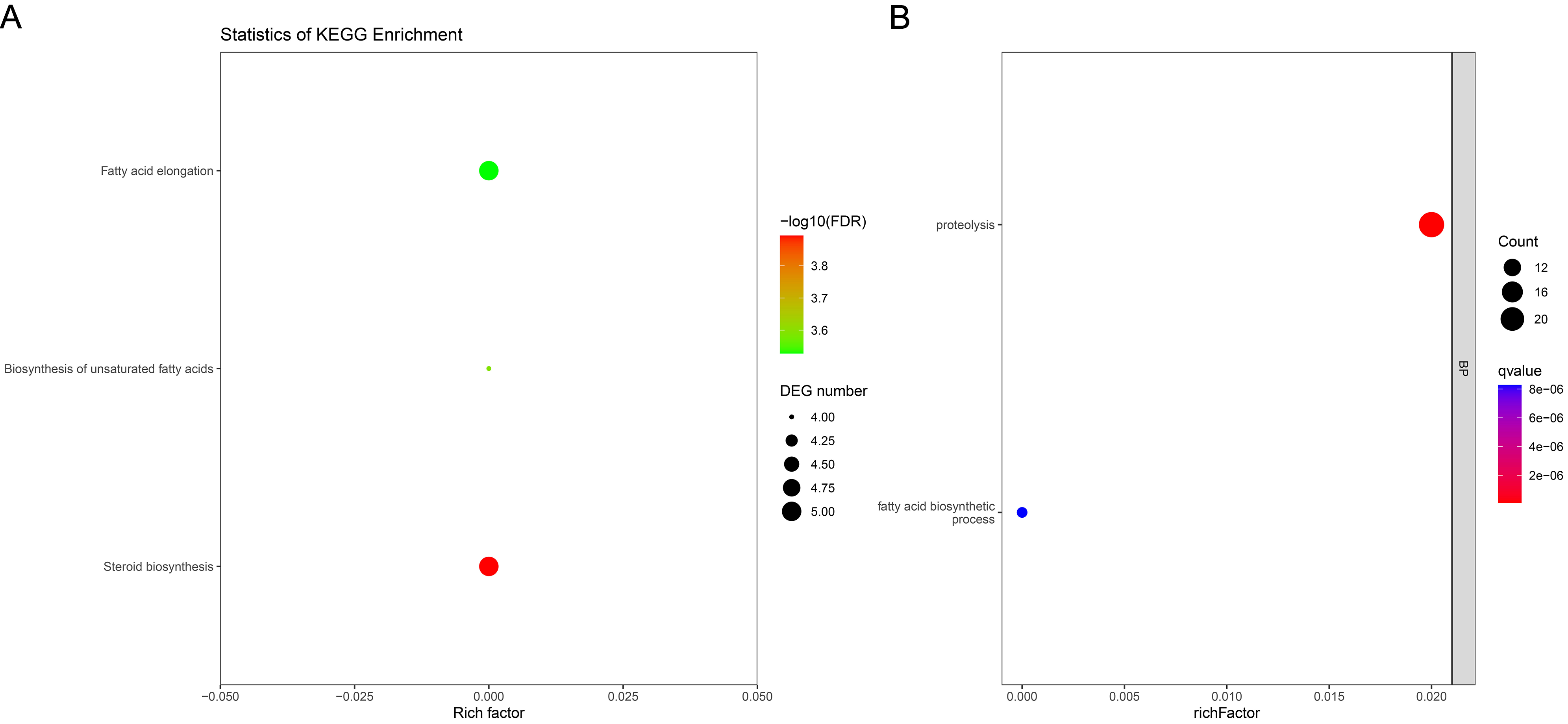

Supplement: Supplementary file 1 [file insects-15-00992-s001.zip › Figure S6 KEGG pathway enrichment analyses (A) and GO function analyses (B) of genes in cluster E.tif]
